# Ferromagnetic resonance spectra of linear magnetosome chains

**DOI:** 10.3762/bjnano.15.15

**Published:** 2024-02-05

**Authors:** Elizaveta M Gubanova, Nikolai A Usov

**Affiliations:** 1 National Research Nuclear University “MEPhI”, 115409, Moscow, Russia; 2 Pushkov Institute of Terrestrial Magnetism, Ionosphere and Radio Wave Propagation, Russian Academy of Sciences, IZMIRAN, 108480, Troitsk, Moscow, Russia

**Keywords:** chains of magnetosomes, ferromagnetic resonance spectra, magnetite nanoparticles, numerical simulation

## Abstract

The ferromagnetic resonance (FMR) spectra of oriented and non-oriented assemblies of linear magnetosome chains are calculated by solving the stochastic Landau–Lifshitz equation. The dependence of the shape of the FMR spectrum of a dilute assembly of chains on the particle diameter, the number of particles in a chain, the distance between the centers of neighboring particles, the mutual orientation of the cubic axes of particle anisotropy, and the value of the magnetic damping constant is studied. It is shown that FMR spectra of non-oriented chain assemblies depend on the average particle diameter at a fixed thickness of the lipid magnetosome membrane, as well as on the value of the magnetic damping constant. At the same time, they are practically independent of the number *N*_p_ of particles in the chain under the condition *N*_p_ ≥ 10. The FMR spectra of non-oriented assemblies of magnetosome chains are compared with that of random clusters of interacting spherical magnetite nanoparticles. The shape of FMR spectra of both assemblies is shown to differ appreciably even at sufficiently large values of filling density of random clusters.

## Introduction

Magnetotactic bacteria are living organisms that grow within themselves magnetite nanoparticles called magnetosomes [1–4]. In contrast to chemically synthesized magnetite nanoparticles [5–6], magnetosomes have a perfect crystal structure, a narrow size distribution, and a high saturation magnetization close to that of bulk magnetite. In particular, magnetotactic bacteria *M. gryphiswaldense* produce linear chains of quasi-spherical magnetite nanoparticles with sizes ranging from 30 to 50 nm [1–2,7–9]. However, there are also magnetotactic bacteria that produce elongated magnetite nanoparticles [1–2,10–11].

A linear chain of uniformly magnetized magnetosomes grown inside a magnetotactic bacterium is a kind of magnetic needle that helps the bacterium navigate in the weak Earth's magnetic field in search of the best habitat [1–4]. Chains of magnetosomes are frequently found in weakly magnetized fossil rocks and bottom sediments, the study of which provides valuable information about the geological and biological past of the Earth [3–4,12–13]. Magnetosome assemblies are very promising also for application in biomedicine [3,5]. The properties of magnetic nanoparticle assemblies are often characterized by measuring ferromagnetic resonance (FMR) spectra [14–15]. The analysis of FMR spectra makes it possible to determine the effective magnetic field in the sample under study, which depends on the particle saturation magnetization, the type of magnetic anisotropy, the direction of the particle easy anisotropy axes, and other parameters. In addition, the FMR spectrum is sensitive to the presence of magnetostatic interactions in dense assemblies of magnetic nanoparticles. Thus, ferromagnetic resonance spectroscopy is a promising technique to study magnetic properties of magnetosome assemblies [7–9,16–23]. However, the FMR spectra depend on many magnetic and geometric parameters of the nanoparticles. Therefore, the interpretation of FMR spectra is a non-trivial problem [16–17,20–25]. For correct interpretation of the FMR spectra, it is highly desirable to use the results of detailed micromagnetic modeling, which takes into account the main physical factors affecting the FMR spectra, including the effect of strong magnetic dipole interactions in magnetosome chains.

Both magnetosomes grown in the laboratory by various types of magnetotactic bacteria [7–9,16–19] and particles found in natural samples of silt and lake sediments [16–17,20–23] have been experimentally studied. It is important that the experimental FMR spectra of magnetosome chains have characteristic differences from those of assemblies with a random arrangement of nanoparticles in the sample [7,16–23]. This helps to detect the presence of magnetosome chains in a natural sample, which is important for paleomagnetic studies. Nevertheless, the problem of comparing the FMR spectra of magnetosome chains and random assemblies of magnetite nanoparticles is far from a complete solution and requires further investigation.

Note that the theoretical description of FMR spectra of assemblies of magnetosome chains is carried out, as a rule, on the basis of simplified models [22–25], in which the behavior of a magnetosome chain in an alternating (ac) high-frequency magnetic field is replaced by the behavior of a uniformly magnetized ellipsoid with an appropriately selected demagnetizing factor. As a result, important information about the internal geometry of the chain, that is, about the particle diameters, the number of particles in the chain, the characteristic distance between the particle centers, and the mutual orientation of the particle cubic anisotropy axes, is completely lost. In addition, in the approach [22–25], only the position of the resonance peak is actually calculated, whereas the shape of the resonance curve is assumed to be Lorentzian or Gaussian, the width of the curve being an adjustable parameter. Obviously, based on such a simplified model, it is practically impossible to obtain information about the internal geometry of the chain and a number of particle magnetic parameters.

It has been shown recently [26–28] that the true geometry of the magnetosome chains has a great influence on the magnetostatic properties of the chain assembly. In this regard, it should be noted that the correct calculation of the FMR spectra of magnetic nanoparticle assemblies can be carried out by solving the stochastic Landau–Lifshitz equation [29–35]. This approach makes it possible, when calculating the FMR spectra, to take into account all the details of the geometric structure of magnetosome chains, the influence of strong magnetic dipole interactions between the particles of the chain, as well as the effect of thermal fluctuations of magnetic moments of nanoparticles at a finite temperature.

Using this approach, in this paper the FMR spectra of oriented assemblies of linear chains of quasi-spherical magnetosomes are calculated depending on the direction of the external magnetizing field with respect to the common axis of the chains; the FMR spectra of randomly oriented assemblies were obtained by the corresponding angle averaging. Various types of mutual orientation of cubic easy anisotropy axes of the chain particles are considered. The FMR spectra of randomly oriented assemblies of magnetosome chains are compared with that of random clusters of interacting spherical magnetite nanoparticles. The theoretical results obtained seem to be helpful for correct interpretation of the large amount of experimental data [1–4,7–9,16–23] accumulated to date for assemblies of magnetosome chains.

## Numerical Simulation

Consider a dilute assembly of linear chains of magnetosomes consisting of *N*_p_ spherical nanoparticles of average diameter *D*. Dynamics of the unit magnetization vector 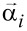 of the *i*-th single-domain nanoparticle of the chain is governed by the stochastic Landau–Lifshitz equation [29–32],


[1]

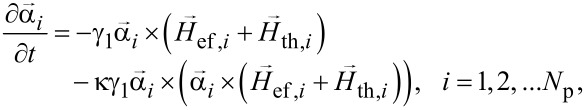



where γ is the gyromagnetic ratio, γ_1_ = γ/(1 + κ^2^), κ is the magnetic damping constant, 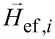 is the effective magnetic field, and 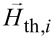 is the thermal field. The effective magnetic field acting on a separate nanoparticle can be calculated as a derivative of the total chain energy *W* = *W*_mc_ + *W*_md_ + *W*_Z_:


[2]

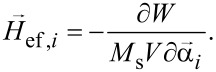



The cubic magneto-crystalline anisotropy energy of Fe_3_O_4_ nanoparticles is


[3]

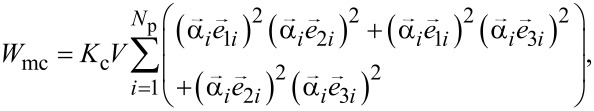



where *V* = π*D*^2^/6 is the volume of spherical particle, *K*_c_ is the cubic anisotropy constant, and (***e***_1_*_i_*, ***e***_2_*_i_*, ***e***_3_*_i_*) is the set of orthogonal unit vectors that determine the spatial orientations of the cubic easy anisotropy axes of the *i*-th nanoparticle of the chain.

For nearly spherical uniformly magnetized nanoparticles, the magnetostatic energy of the chain can be represented as the energy of the point interacting dipoles located at the particle centers ***r****_i_*


[4]

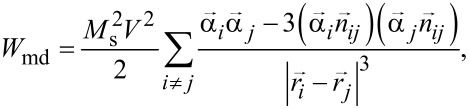



where ***n****_ij_* is the unit vector along the line connecting the centers of the *i*-th and *j*-th particles.

The Zeeman energy of the assembly in a applied magnetic field ***H*** and a weak perpendicular ac magnetic field ***H***_1_sin(ω*t*) is given by


[5]

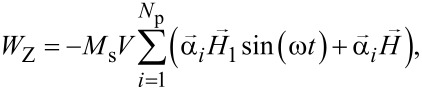



where ω = 2π*f* is the angular frequency of the ac magnetic field.

The thermal fields 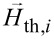 acting on various nanoparticles of the chain are statistically independent, with the following statistical properties [29] of their components


[6]

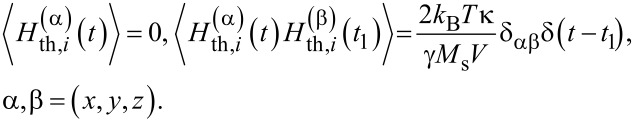



Here *k*_B_ is the Boltzmann constant, δ_αβ_ is the Kroneker symbol, and δ(*t*) is the delta function.

It is well known [14–15,33–35] that the power absorbed by the assembly per unit time and per unit volume is proportional to the area of the assembly hysteresis loop


[7]

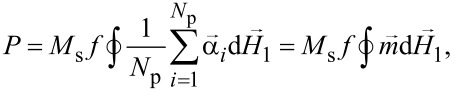



where ***m*** is the reduced magnetic moment of the assembly. To numerically calculate the power absorbed by an assembly of superparamagnetic nanoparticles in ac magnetic field ***H***_1_(*t*), it is convenient to rewrite Equation 7 in the form of the time-averaged integral


[8]

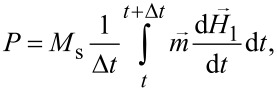



where Δ*t* is a certain time interval significantly exceeding the period of oscillations of the ac magnetic field, τ = 2π/ω. Using the small amplitude of the ac magnetic field, the same quantity can be expressed in terms of the imaginary part of the magnetic susceptibility of the assembly [15,36],


[9] $$P=\pi f\chi^{\prime \prime }\left(H,f\right)H_{1}^{2}.$$

Comparison of Equation 8 and Equation 9 makes it possible to obtain the imaginary part of the magnetic susceptibility χ″(*H*,*f*) of the assembly as a function of the magnetizing field ***H***.

In this paper the calculation of the specific absorbed power is carried out using Equation 8 for dilute assemblies of linear chains of magnetosomes with saturation magnetization *M*_s_ = 460 emu/cm^3^ and cubic magnetic anisotropy constant *K*_c_ = −1.1 × 10^5^ erg/cm^3^ [37]. The average diameter of particles in a chain varies in the range *D* = 20–45 nm, the number of nanoparticles in a chain is *N*_p_ = 5–30, and the magnetic damping constant is taken as κ = 0.05–0.5. The frequency of ac magnetic field exciting the resonance is *f* = 4.9 GHz (S band) or *f* = 9.8 GHz (X band), the amplitude of a weak ac magnetic field is *H*_1_ = 10 Oe.

For completeness of the study, we considered several characteristic types of mutual orientation of the cubic anisotropy axes of the nanoparticles in a chain. For the case of a completely random orientation of the cubic axes (index *R*), the set of orthogonal unit vectors (***e***_1_*_i_*, ***e***_2_*_i_*, ***e***_3_*_i_*), *i* = 1,2, …, *N*_p_ is randomly oriented in each nanoparticle. The case when one of the cubic easy anisotropy axes of each particle is parallel to the chain axis is denoted below by the index *E*. In this case, the directions of the other cubic anisotropy axes of the particles are randomly oriented. Similarly, the case when one of the hard axes of cubic anisotropy is parallel to the chain axis, while the other axes are randomly oriented, is denoted below by the index *H*.

To obtain statistically significant results, the FMR spectra of a chain assembly are averaged over a sufficiently large number of independent numerical experiments, *N*_exp_ = 20–30. In each experiment, a new linear chain of *N*_p_ interacting spherical magnetite nanoparticles was created, the directions of the cubic anisotropy axes of particles being oriented according to the accepted chain anisotropy.

When solving the stochastic Landau–Lifshitz equation, the numerical time step is kept to be 1/30 of the characteristic precession period of the unit magnetization vectors of the particles, τ_H_ ~ 1/γ_1_*H*_ef_. Such a small time step is necessary to accurately describe the precession of unit magnetization vectors in the chain. In addition, the total time interval of the calculation covered at least 200 periods of the ac magnetic field, while the time averaging of the integral in Equation 8 occurred only over the last quarter of the total number of periods, when the dynamics of the unit magnetization vectors of the particles became stationary. Thus, the time interval Δ*t* in Equation 8 exceeds 50 periods of the ac magnetic field. Averaging the numerical results for the absorbed power of the assembly over a sufficiently long time interval Δ*t* and over a fairly representative set *N*_exp_ of independent realizations of chains with a fixed number of nanoparticles and type of chain anisotropy accepted makes it possible to obtain statistically significant results for the magnetic susceptibility of a dilute assembly of linear chains.

## Results and Discussion

An analysis of transmission electron microscope (TEM) images [1–2,8,38–39] shows a fairly large variability in the geometry of magnetosome chains created by bacteria of various strains. Namely, the average diameter of particles, the characteristic distance between their centers, and the average number of particles in a chain change depending on the bacteria strains. Therefore, it is important to study the dependence of the FMR spectra on the specified chain geometric parameters. In this paper, we restrict ourselves to detailed modeling of the FMR spectra of chains of quasi-spherical magnetosomes with diameters in the range *D* = 20–45 nm. An important geometrical parameter of the chain is also the average distance *a* between the centers of particles in the chain, since this distance determines the amplitude of the dipole field *H*_dip_, acting between the particles of the chain. Based on the TEM data [1–2,7–8,38–39], it can be concluded that the nearest distance between the surfaces of neighboring spherical particles is the sum of the thicknesses of the magnetosome shells 2*T*_en_, where *T*_en_ = 4–6 nm is the characteristic thickness of the lipid magnetosome shell. The latter, apparently, weakly depends on the nanoparticle diameter. If this hypothesis is correct, then the average distance between the particle centers in a chain is *a* = *D* + 2*T*_en_.

When modeling the FMR spectra of magnetosome chains, it is important to choose the adequate magnetic damping constant κ of the magnetic nanoparticles. Unfortunately, experimental data for this quantity for assemblies of magnetic nanoparticles are scarce [40]. Because of the well-known perfection of the crystal structure and shape of magnetosomes, in this paper most of the calculations are carried out for the case of moderate damping, κ = 0.05–0.1; however, the case of high damping, κ = 0.3, 0.5, is also briefly considered. Note that it is experimentally possible [9,41] to create dilute assemblies of magnetosome chains oriented in one direction in a strong external magnetic field. This makes it possible to obtain FMR spectra for oriented assemblies of magnetosome chains depending on the angle of an external magnetic field with respect to the common orientation axis of the chains [9]. As will be shown below, the FMR spectra of oriented chain assemblies strongly depend on the specific geometric structure and magnetic characteristics of the magnetosomes.

In this work, we first calculate the FMR spectra of oriented assemblies of chains as a function of the angle θ of the external magnetic field with respect to the orientation axis of the chains. The spectra of randomly oriented assemblies of chains were then calculated by the angle averaging of partial FMR spectra calculated with a fairly small step Δθ ~ 5–7.5°. Further, we discuss the effect of geometric and magnetic parameters on the FMR spectra of oriented and non-oriented dilute assemblies of chains of quasi-spherical magnetosomes. The numerical results obtained are presented as dependences of the magnetic susceptibility of a chain assembly on its geometric and magnetic parameters, since the magnetic susceptibility is a fundamental physical quantity that characterizes the magnetic properties of the assembly. However, the derivatives of the magnetic susceptibility are also given in some cases for comparison.

**Figure 1:**
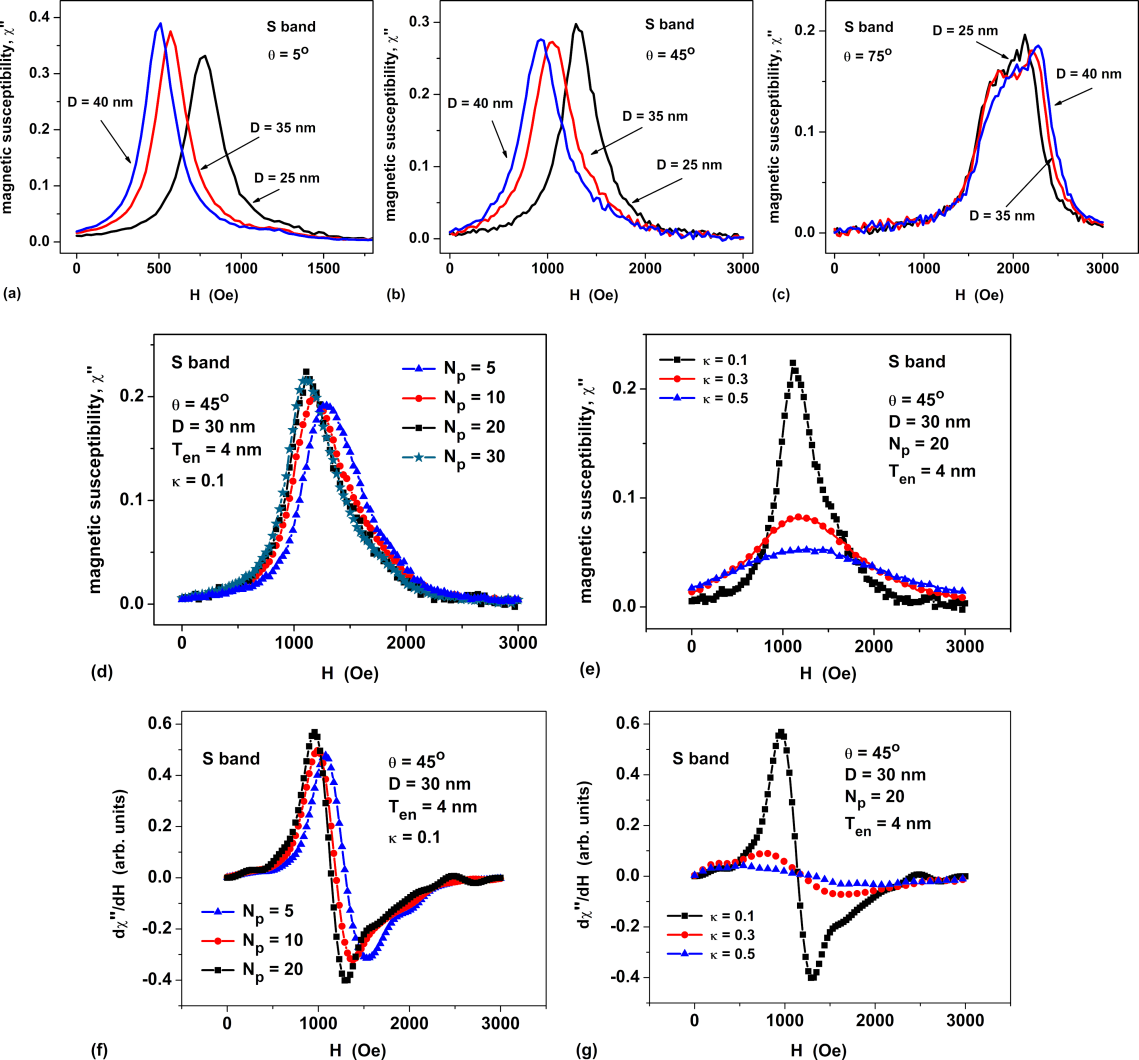
(a–c) Comparison of FMR spectra of oriented chains of magnetosomes with different particle diameters *D* for angles θ = 5°, 45° and 75°, respectively. The number of particles in the chain is *N*_p_ = 20, the magnetic damping constant is κ = 0.1. (d) The influence of the FMR spectrum on the number of particles in the chain *N*_p_. (e) Dependence of the FMR spectra on the value of the magnetic damping constant κ. The thickness of the lipid membrane of magnetosomes is *T*_en_ = 4 nm, the frequency of the ac magnetic field is *f* = 4.9 GHz (S band). (f, g) Derivatives of the magnetic susceptibility for panels (d) and (e), respectively. The chain anisotropy is of type *E* for Figure 1a–c and type *R* for Figure 1d–g, consequently.

Figure 1a–c shows the dependence of the FMR spectra of an oriented assembly of chains on the particle diameter *D* at a fixed thickness of the lipid shell *T*_en_ = 4 nm and at different directions of the magnetizing field with respect to the orientation axis of the assembly, θ = 5°, 45° and 75°, respectively. As shown in Figure 1a,b, the dependence of the position of the resonance peak on the particle diameter is most pronounced at angles θ ≤ 45°, but it becomes insignificant at θ > 75°. For example, according to Figure 1a, at θ = 5° the resonance peak for chains with diameter *D* = 25 nm occurs at *H* = 760 Oe, while for chains with *D* = 40 nm the resonance field is much lower, *H* = 530 Oe. To explain this effect, it is worth noting that the dipole field in the middle part of a long chain magnetized along its axis can be estimated as *H*_dip_ = (2π/3)*M*_s_ ζ(3)/(1 + 2*T*_en_/*D*)^3^, where ζ(3) ≈ 1.2 is the value of the Riemann zeta function [42]. With a shell thickness *T*_en_ = 4 nm, from this formula one obtains *H*_dip_ = 503 Oe at *D* = 25 nm, and *H*_dip_ = 670 Oe for *D* = 40 nm, respectively. Thus, for small angles θ the dipole field acting along the chain axis decreases as a function of particle diameter. Accordingly, the FMR peak for a chain of particles of smaller diameter should be observed at a larger magnetizing field.

As Figure 1d shows, with an increase in the number of particles in the chain from 5 to 20, the position of the FMR resonance peak shifts to lower fields; however, for *N*_p_ > 20 the position and shape of the FMR resonance peak remain practically unchanged. Calculations show that the characteristic value of the dipole field *H*_dip_ stabilizes already on the first two or three periods of the chain and does not depend on its length. According to the experimental data [1,7,19], in the chains of *M. gryphiswaldense* bacteria the characteristic number of magnetosomes is *N*_p_ = 20–25. Therefore, in this work most of the calculations were carried out for magnetosome chains with *N*_p_ = 20.

As noted above, the experimental data on the value of the magnetic damping constant in assemblies of magnetic nanoparticles are scarce. Since magnetosomes grow inside a bacterium under strict genetic control, they turn out to be the perfect magnetic particles in terms of their crystal structure and shape. Therefore, it is reasonable to assume that the magnetic damping constant for magnetosomes takes relatively small values, κ ≤ 0.1. These values were mainly used in the calculations. However, the value of the magnetic damping constant has a strong influence on the shape of the FMR spectrum peak. As Figure 1e shows, as κ increases, the position of the FMR peak does not change, but its height decreases significantly, while the peak width increases. Figure 1f and Figure 1g, which show the derivatives of magnetic permeability with respect to the magnetic field, confirm the above conclusions about the influence of the number of particles in linear chains and the value of the magnetic damping constant on the FMR spectrum.

There are convincing arguments [41] that *E*-type anisotropy is realized in magnetosome chains as a rule. This means that one of the equivalent cubic easy anisotropy axes of every particle is parallel to the chain axis. It is argued [41] that as the chain grows, new magnetosomes sequentially appear at the ends of the chain, and their formation occurs in a strong dipole field directed along the chain axis. In contrast, it was shown [39] that the formation of magnetosomes in a bacterium can occur simultaneously in many germ vesicles along its length. In this case, it is not clear what reason can lead to the occurrence of *E*-type anisotropy in the chain. Rather, one would expect a random orientation of the cubic anisotropy axes of individual nanoparticles, that is, the formation of *R*-type chain anisotropy. Note that different types of chain anisotropy can be modeled by choosing properly the orientation of the reference vectors (***e***_1_*_i_*, ***e***_2_*_i_*, ***e***_3_*_i_*) of individual nanoparticles of the chain in Equation 2.

**Figure 2:**
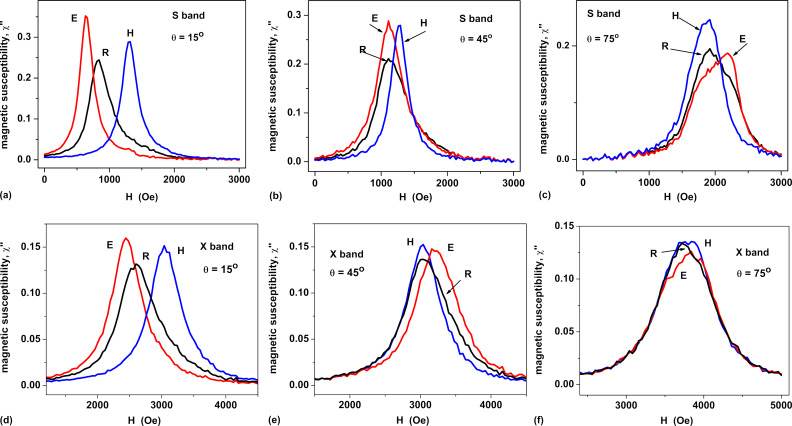
Dependence of the FMR spectra of an oriented assembly of chains on the relative orientation of the cubic anisotropy axes in the particles of the chain for different directions of the magnetizing field and various resonance frequencies. (a–c) S band and (d–f) X band. Indexes mark various types of chain anisotropy: *R* corresponds to random chain anisotropy, *E* means that one of the cubic easy axes is parallel to the chain axis, and *H* indicates that one of the hard axes is parallel to the chain axis.

In Figure 2a–c we compare the S-band FMR spectra of dilute oriented assemblies of magnetosome chains with different mutual orientations of the cubic anisotropy axes of individual particles within the chain for some directions of the magnetizing field. The number of particles in chains *N*_p_ = 20, the particle diameter is *D* = 40 nm, the membrane shell thickness is *T*_en_ = 4 nm, and the magnetic damping constant is κ = 0.1. As Figure 2 shows, the greatest difference in the position of the FMR peaks for chains with different types of anisotropy is observed at angles θ ≤ 15°. In addition, the height of the FMR peak for chains with random anisotropy turns out to be noticeably smaller than that for *E*- and *H*-type anisotropies. Thus, the type of chain anisotropy can have an effect on the shape of the FMR spectrum of an assembly of magnetosome chains.

For comparison, Figure 2d–f represents the X-band FMR spectra of the same dilute assemblies of linear magnetosome chains. It can be noted that for the X band the amplitude of the FMR peaks is on average half of that for the S band. In addition, for the X FMR band the peaks of assemblies with anisotropy types *R*, *E*, and *H* are poorly resolved for angles θ ≥ 75°.

**Figure 3:**
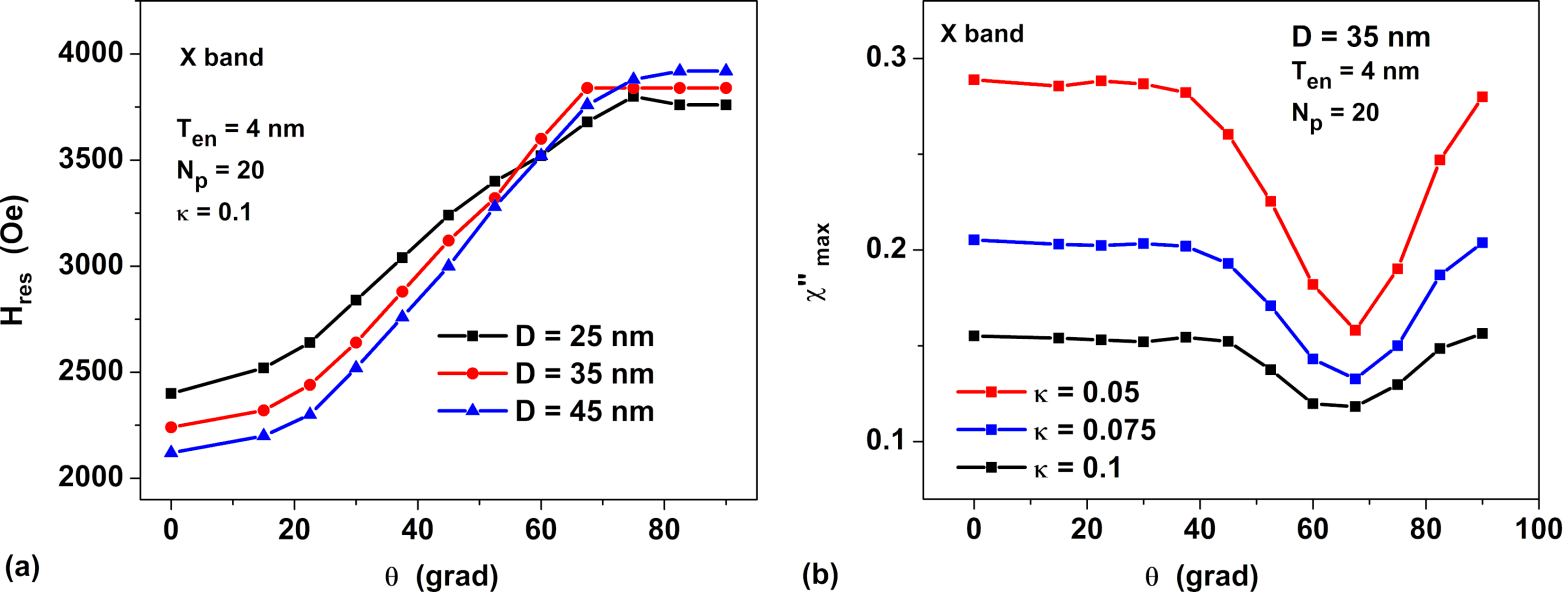
(a) The resonance FMR field as a function of polar angle θ for oriented assemblies of linear magnetosome chains with various particle diameters at a fixed value of magnetic damping constant κ = 0.1. (b) Magnetic susceptibility peak strength as a function of polar angle θ for oriented assemblies of chains with different damping constants at a fixed particle diameter *D* = 35 nm. The chain anisotropy is of type *E.*

To summarize the numerical simulation data obtained for oriented assemblies, in Figure 3a we show the angle dependence of the resonance FMR field, *H*_res_(θ), for linear chains with various average particle diameters. It is interesting to note the noticeable dependence of the position of the resonance peak on the particle diameter *D* at small polar angles θ ≤ 20–25°. In addition, Figure 3b shows that the average height of the resonant FMR peaks of an oriented chain assembly depends significantly on the magnetic damping constant. These findings make it possible to estimate average particle diameter and damping constant from comparison of experimental and theoretical data.

**Figure 4:**
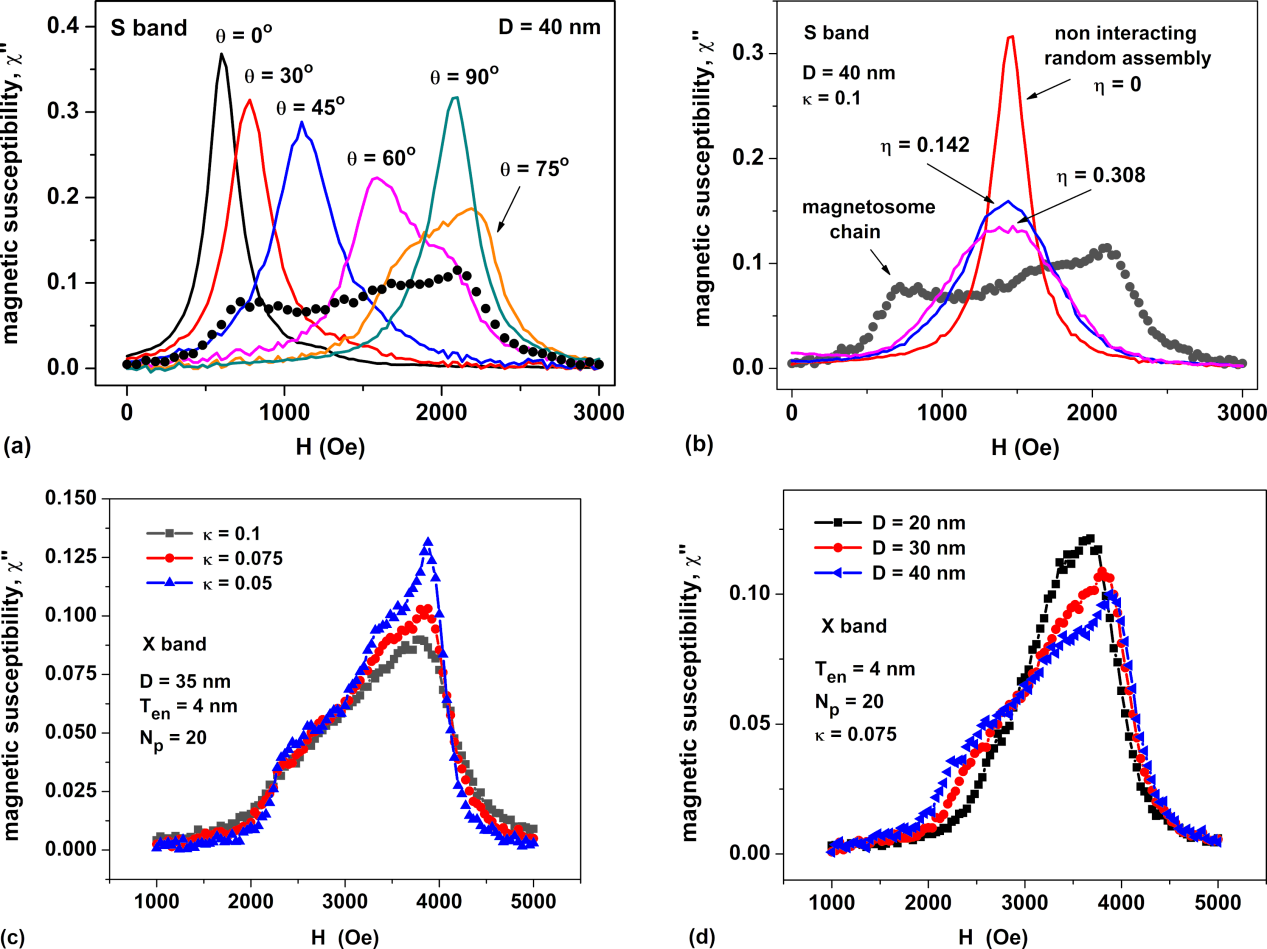
(a) Formation of the FMR spectrum of a non-oriented assembly of magnetosome chains (black dots) as a result of angle averaging of partial FMR spectra of oriented assemblies calculated for various θ angles (solid color curves). (b) Comparison of the FMR spectrum of a non-oriented assembly of magnetosome chains with the FMR spectra of a random assembly of magnetite clusters with different filling density η. (c, d) Dependence of the magnetic susceptibility of a non-oriented chain assembly on the damping constant κ at a fixed magnetosome diameter *D* = 35 nm, and on the average magnetosome diameter at a fixed value of κ = 0.075, respectively. Figure 4a,b: S band, Figure 4c,d: X band.

Let us now turn to the description of the FMR spectra of dilute non-oriented assemblies of magnetosome chains. The latter were obtained by averaging partial FMR spectra of oriented assemblies over the polar angle θ. Note that the FMR spectra of chains are averaged over the declination φ even at the stage of calculating the FMR spectra of oriented assemblies, since for any type of chain anisotropy, the orientation of the cubic anisotropy axes in directions perpendicular to the chain axis is random. The calculations presented in Figure 4 are carried out for the chain anisotropy of type *E*, since this anisotropy type prevails [41], apparently, for quasi-spherical magnetosomes of *M. gryphiswaldense*.

Figure 4a explains the formation of the FMR spectrum of a non-oriented assembly of magnetosome chains with *E*-type anisotropy (black dots) upon averaging partial FMR spectra of oriented assemblies over the angle θ. Partial FMR spectra at certain angles θ are shown in Figure 4a as solid colored curves. The number of particles in chains is *N*_p_ = 20, the particle diameter is *D* = 40 nm, the membrane shell thickness is *T*_en_ = 5 nm, and the magnetic damping constant is κ = 0.1. To obtain FMR spectra of randomly oriented chain assemblies, partial FMR spectra were averaged with a step width of Δθ = 5°. As Figure 4a shows, with an increase in the tilt angle of the magnetizing field θ from 0° to 75°, the peak of resonant absorption of the oriented assembly of chains shifts towards higher field values. Simultaneously, the peak height decreases.

The shift in the position of the resonant peak of the oriented assembly is a consequence of the weakening of the component of the dipole field *H*_dip_, which acts in the direction of the magnetizing field *H*, when the magnetic moments of the particles deviate from the axis of the chain. However, as the angle increases, θ > 75°, when the magnetic moments of nanoparticles in a sufficiently strong magnetizing field become almost perpendicular to the chain axis, the position of the FMR peak does not shift further, whereas the magnetic susceptibility of the assembly increases. As Figure 4a shows, because of the angle dependence of partial FMR peaks, the FMR peaks of non-oriented assemblies of magnetosome chains are much wider than the width of the peaks of the individual partial FMR spectra. This is a characteristic property of the FMR spectra of non-oriented assemblies of magnetosome chains, which distinguishes them from FMR spectra of random assemblies of magnetite nanoparticles.

To confirm this conclusion, in Figure 4b we compare the FMR spectrum of the non-oriented assembly of chains shown in Figure 4a with the FMR spectra of a random assembly of clusters of interacting magnetite nanoparticles calculated for different cluster filling densities η = *N*_p_*V*/*V*_cl_. Here, *V*_cl_ is the volume of a random cluster containing *N*_p_ = 60 spherical magnetite nanoparticles of the same diameter *D* = 40 nm, randomly located in the cluster volume and having a random orientation of the cubic anisotropy axes. As Figure 4b shows, the width of the FMR spectra of dilute assemblies of random clusters increases with an increase in the filling density η because of an increase in the intensity of the magnetic dipole interactions within the clusters. For example, at η = 0.308, when the average distance between particle centers in a dense random cluster is rather small, *L* = (π/6η)^1/3^*D* ≈ 1.2*D*, the width of the FMR spectrum of the assembly of random clusters at half maximum is approximately Δ*H* = 1000 Oe. Nevertheless, the width of FMR spectra of non-oriented assemblies of chains at half maximum is much wider than this. It is approximately given by Δ*H* = 1800 Oe. In addition, the shape of FMR spectra of assemblies of non-oriented chains differs from the spectra of assemblies of random clusters by the presence of two local peaks at *H* = 750 Oe and *H* = 2250 Oe.

In Figure 4c we compare the FMR spectra of assemblies of non-oriented magnetosome chains with fixed diameter *D* = 35 nm for various values of the magnetic damping constant κ = 0.05, 0.075, and 0.1. Obviously, with decreasing κ, the height of the magnetic susceptibility peak of the assembly increases, while the peak width somewhat decreases. In contrast, as Figure 4d shows that at a fixed value of κ the height of the magnetic susceptibility peak decreases with increasing particle diameter. Note that the absorption peaks in Figure 4c and Figure 4d are shifted to the right, since in a non-oriented assembly of chains the probability of finding a chain oriented at angle θ to the magnetizing field direction is proportional to sinθ dθ. It is an increasing function of θ in the range 0 < θ < 90°.

Based on the data given in Figure 4c and Figure 4d, one can conclude that the FMR spectra of assemblies of non-oriented chains of magnetosomes depend significantly on the value of the magnetic damping constant κ; at a fixed thickness of the lipid shell of magnetosomes, they depend on the average particle diameter *D*. At the same time, the calculations performed show that the dependence of the FMR spectra of non-oriented assemblies of chains is practically independent of the number of particles in the chain under the condition *N*_p_ ≥ 10.

**Figure 5:**
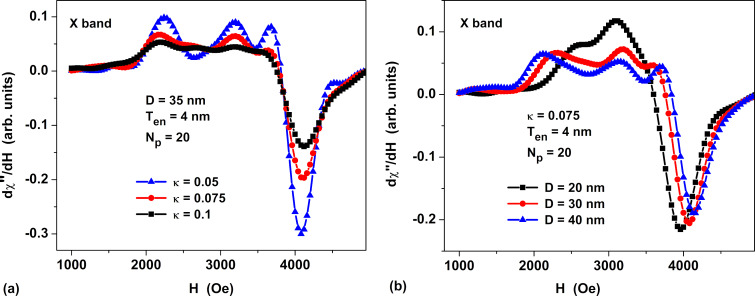
Derivatives of the magnetic susceptibility with respect to the magnetizing field for non-oriented assemblies of linear chains of quasi-spherical magnetosomes. (a) Dependence of the spectra on the magnetic damping constant at a fixed average nanoparticle diameter *D* = 35 nm and (b) dependence of the spectra on the nanoparticle diameter at magnetic damping constant κ = 0.075.

Figure 5 shows the derivatives of the magnetic susceptibility with respect to the magnetizing field for non-oriented assemblies of linear chains of quasi-spherical magnetosomes. Spectra of this type are usually measured in ferromagnetic resonance experiments on assemblies of magnetic nanoparticles [7–9,16–23]. As Figure 5a shows, the depth of the sharp negative peak at *H* ≈ 4000 Oe, which is typical for non-oriented assemblies of linear chains of magnetosomes [7–9,16–18], depends significantly on the value of the magnetic damping constant. At the same time, according to Figure 5b, the position of this negative peak depends on the average diameter of the nanoparticles in the chain.

## Conclusion

In this paper the FMR spectra of oriented and non-oriented assemblies of magnetosome chains are calculated by solving the stochastic Landau–Lifshitz equation. Calculations of the imaginary component of the high-frequency magnetic susceptibility of assemblies in a magnetizing field are carried out with a small time step, which is 1/30 of the characteristic precession time of the particle unit magnetization vectors. In addition, the power absorbed by the assembly is averaged over a sufficiently large number of periods of the ac magnetic field because of the stochastic dynamics of the unit magnetization vectors. This makes it possible to obtain statistically reliable results for the high-frequency magnetic susceptibility of a dilute assembly of linear chains of magnetosomes.

In this paper, in contrast to the simplified models [22–25], it is shown that using the solution of the stochastic Landau–Lifshitz equation one can take into account all the important details of the geometric structure of magnetosome chains that significantly affect the shape of the FMR spectrum of a chain assembly. For a fixed thickness of the lipid membrane of magnetosomes the FMR spectra of both oriented and non-oriented chain assemblies are shown to depend on the average particle diameter. However, the dependence of the FMR spectra on the number of particles in the chain appears only for short, dangling chains, with the number of particles *N*_p_ < 10. We also studied the dependence of the FMR spectra of oriented chain assemblies on the mutual orientations of the cubic easy anisotropy axes of particles along the chain. It is found that for chains with a random orientation of the cubic easy axes the height of the FMR peak is noticeably smaller than that for the other anisotropy types considered. It is also found that the FMR spectrum of a chain assembly essentially depends on the value of the phenomenological magnetic damping constant. Finally, the FMR spectra of non-oriented assemblies of magnetosome chains were compared with the FMR spectrum of a dilute assembly of random clusters of spherical nanoparticles with different cluster filling density η. With an increase in η, that is, with an increase in the intensity of the magnetic dipole interactions in the clusters, the width of the FMR peak of an assembly of random clusters increases significantly. Nevertheless, it remains much smaller than the peak width of an assembly of chains even for very dense clusters with η = 0.308, when the average distance between the particle centers in the cluster is only *L* ≈ 1.2 *D*. The shape of the FMR spectra for the two types of assemblies considered also differs. The information obtained in this paper may help improve the interpretation of the FMR spectra of various assemblies of magnetic nanoparticles.
